# Introduction and scientific justification of data transportability for confined field testing for the ERA of GM plants

**DOI:** 10.3389/fbioe.2024.1359388

**Published:** 2024-02-21

**Authors:** Shuichi Nakai, Andrew F. Roberts, Abigail R. Simmons, Kazuyuki Hiratsuka, Douglas W. Miano, Facundo Vesprini

**Affiliations:** ^1^ Bayer CropScience K.K., Tokyo, Japan; ^2^ International Life Science Institute Japan, Tokyo, Japan; ^3^ Agriculture & Food Systems Institute, Washington, DC, United States; ^4^ CropLife International, Arlington, VA, United States; ^5^ Graduate School of Environment and Information Sciences, Yokohama National University, Yokohama, Japan; ^6^ Department of Plant Science and Crop Protection, University of Nairobi, Nairobi, Kenya; ^7^ Bayer CropScience, Buenos Aires, Argentina

**Keywords:** data transportability, genetically modified plant, environmental risk assessment, confined field testing, problem formulation

## Abstract

The concept of Data Transportability (DT) of Confined Field Testing (CFT) to support the Environmental Risk Assessment (ERA) of Genetically Modified (GM) plants was first introduced in the literature by Garcia-Alonso et al., in 2014. Since then, DT has been discussed in many countries and regions as a concept to prevent duplication of regulatory studies without compromising quality of the ERA. However, despite its usefulness and scientific justification, DT is not well adopted at this time and many regulatory agencies around the world require additional in-country CFT be conducted before approving GM plants. Based on the current circumstances, the authors organized a parallel session entitled “*Introduction and Scientific Justification of DT for CFT for the ERA of GM plants*” at 16th ISBR (the International Society for Biosafety Research). This session mainly consisted of the following three parts. The first two speakers, Andrew Roberts and Abigail Simmons provided an overview of DT and examples of conditions for the transportability of field data/conclusions advocated in the peer-reviewed scientific journals. Next, the current status of DT adoption in some countries/regions such as Japan and Africa, and a theoretical case study for Argentina were introduced by Kazuyuki Hiratsuka, Douglas Miano, and Facundo Vesprini, respectively. Lastly, a risk hypothesis-based approach for DT which was developed in advance by the five speakers of this parallel session, was introduced. During the discussion, there was a common understanding that transition to the risk hypothesis-based approach for DT was scientifically appropriate, considering the accumulated evidences that several countries have conducted confirmatory local CFT for more than 20 years but they have not detected any differences related to the ERA assessment endpoints in GM crops. The risk hypothesis-based approach for DT introduced here is expected to play an important role in discussions on the implementation of DT in various parts of the world in the future.

## Graphical Abstract

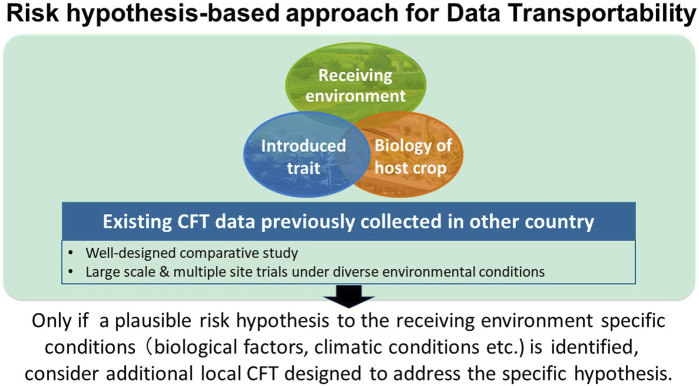

## 1 Introduction

The cultivation area of GM crops worldwide increased from 1.7 million hectares to over 202.2 million hectares from 1996 to 2021, and the number of cultivation countries also increased from 6 to 27 countries (AgbioInvestor, 2023). The majority of GM crops currently grown commercially are soybean, corn, cotton and canola, but other GM crops such as alfalfa, sugar beet, brinjal, rice, sugar cane, wheat, potato, and tomato have also been commercialized (AgbioInvestor, 2023). Since the beginning of commercial cultivation in 1996, GM crops have been rapidly accepted by farmers around the world, mainly due to improved farmer incomes resulting from increased yield and cost reductions, as well as reduced pesticide usage (Brookes, 2022).

Although GM crops are expected to benefit farmers around the world, there are various barriers to adoption. According to a 2022 study from Agbio Investor, which surveyed four leading biotech crop developers (BASF, Bayer, Corteva, and Syngenta), the average time it takes for a new GM crop to be commercialized has increased by about 26% from 13.1 years for a crop launched 2008–2012 to 16.5 years for a crop launched 2017–2022. Additionally, it was revealed that the regulatory phase accounts for 37.6% of the total cost and takes up 51.1% of the nonconsecutive time (AgbioInvestor, 2022). The extra time taken in the regulatory process delays the access of farmers and consumers to innovations in both cultivation and import countries. Another consequence of this situation is that developers in small companies, startups or from the public sector are discouraged from pursuing interesting projects involving GM crops, limiting innovations to large multinational companies and major commodity crops (Lewi and Vicién, 2020).

Global regulatory data requirements that are fit for purpose and hypothesis-driven, based on the application of adequate problem formulation process, would help overcome these barriers and allow valuable innovations to contribute to food security and reduce environmental impact without compromising biosafety. Laboratory and/or field studies on GM crops are conducted as part of the ERA to determine whether cultivation or incidental release of the GM crops could cause environmental harm. Sometimes, even in the absence of country-specific risk hypotheses, regulatory agencies require local laboratory and/or CFT in a country intending to cultivate GM crops. Some agencies also require local agronomic studies when the GM plant products (e.g., grain) is intended only for import, not for cultivation. For example, regulatory agencies in Japan (Ministry of Agriculture, Forestry and Fisheries, MAFF and Ministry of the Environment, MOE) have required local field studies for import approvals for some GM events, depending on the crop (s) and trait (s). The regulatory agency in China (Ministry of Agriculture and Rural Affairs, MARA) accepts global data as part of the import permit application, but local field studies as well as other laboratory-based studies are then commissioned by MARA to be conducted by a local institution in China. The requirements to repeat studies in different countries without identified risk hypotheses leads to duplication of data and add time and complexity to the regulatory process of GM plant without improving the ERA.

DT has been discussed in many countries and regions as a way to prevent the unwarranted duplication of regulatory studies without compromising the quality of the ERA. However, despite its usefulness and scientific justification, DT is not yet fully adopted, as many regulatory agencies around the world require additional in-country CFT be conducted before approving GM plants. There are primarily two factors why DT is not fully adopted. Firstly, in contrast to controlled environment like laboratories or greenhouses, where testing conditions can be consistently regulated, the growth of GM crops in field, like all crops in the field, may exhibit phenotypic variation in different environment, such as different soil types and weather conditions. Consequently, some regulatory authorities advocate the necessity of conducting CFT specific to the receiving environment. Secondly, there is difficulty of amending the guidelines that regulate GM crops once they have been established. The process of amending guidelines typically entails a complex procedure, which includes a public comment period. Consequently, even in the presence of accumulated scientific evidence, the immediate amendment of these guidelines is generally challenging.

As it was revealed that the regulatory phase accounts for 37.6% of the total cost and takes up 51.1% of the nonconsecutive time (AgbioInvestor, 2022), the extra time taken in the regulatory process including this additional in-country CFT delays the access of farmers to this innovation which could improve farmer incomes resulting from increased yield and cost reductions, as well as reduce pesticide usage. Based on the current circumstance, the authors organized a parallel session entitled “*Introduction and Scientific Justification of DT for CFT for the ERA of GM plants*” at 16th ISBR. In this paper, the authors will introduce the results of this parallel session divided into three items: 1. *Overview of DT and examples of conditions for the transportability of field data/conclusion*, 2. *Current status of DT adoption in some countries/regions such as Japan, Africa and Argentina* and, 3. *A risk hypothesis-based approach for DT*.

## 2 Overview of DT and examples of conditions for the transportability of field data/conclusion

Andrew Roberts, CEO of Agriculture & Food Systems Institute (AFSI) and a member of organizing committee of 16th ISBR, gave the first presentation entitled “Introduction of Key Concepts for Data Transportability.” Dr. Roberts explained DT as the ability to use data collected in one geographic region or legal jurisdiction to inform a risk assessment in another region or jurisdiction. He also cited the avoidance of duplication of regulatory efforts as a key benefit of adopting DT. AFSI began studying the conditions for DT in 2011 and published a conceptual framework paper in 2014 (Garcia-Alonso et al., 2014). This paper provides guiding principles for the practical adoption of DT stating that the environmental and agronomic conditions under which the CFT was conducted in the remote country (ies) must be relevant to the conditions in the local country where the GM plant is intended to be cultivated. One way to demonstrate the relevance of environmental conditions is through the use of agroclimate, an aggregate measure of characteristics of the physical environment over time (Melnick et al., 2023). As a tool to visualize similar agroclimate zone, he introduced “Global Environmental Zones Explorer (GEnZ Explorer) (AFSI, 2023). The GEnZ Explorer can provide scientific justification for planning and location of field testing, as well as demonstrating the relevance of previous trials for consideration in another jurisdiction conducting a risk assessment. Dr. Roberts stressed that having a similar agroclimate is one way to demonstrate that an environment has relevance, but it is not a requirement for DT. Rather GEnZ Explorer is intended to provide simple visual information to support scientific justification by risk assessors who need to present a case to decision makers or skeptical public.

Abigail Simmons, Regulatory Manager of CropLife International, gave a second presentation entitled “Data Transportability for Studies Performed to Support an Environmental Risk Assessment for GM crops.” Her presentation was made on the basis of the paper written by Bachman et al. (2021). Dr. Simmons explained the rationale for DT using an ERA framework that relies on problem formulation. Problem formulation refers to the process of developing a testable hypothesis of how a GM plant could affect defined protection goals/values. When an ERA is conducted using problem formulation, a set of data such as the nature of intended traits, the receiving environment and the biology of the unmodified crops is considered first. Additional data should only be requested, if there is a plausible testable hypothesis of a pathway to harm from the GM plant (Anderson et al., 2021). CFT is typically conducted in the country where the GM plant is developed, and specific locations are selected to be representative of the agricultural environments where these crops are grown. The data from these well-designed comparative assessments should be transportable even in the absence of agroclimatic similarity. It was argued that additional data, including local CFT should only be requested when a plausible pathway to harm of a protection goal has been identified as the result of conducting problem formulation.

## 3 Current status of DT adoption in some countries/regions such as Japan, Africa and Argentina

From Japan, Kazuyuki Hiratsuka, Yokohama National University and a Committee member of the ERA under the Japanese Ministry of Agriculture, Forestry and Fishers (MAFF), and Ministry of the Environment (MOE) made a presentation entitled “Data Transportability of GM corn and cotton for familiar traits in Japan” to introduce the current status of ERA and DT in Japan. In introducing the ERA process in Japan, Dr. Hiratsuka emphasized that the committee members who carry out the ERA are made up of experts from various fields with comprehensive expertise. This allows the ERA of GM crops to be conducted from multiple perspectives. Regarding the examples to adopt DT in Japan, Dr. Hiratsuka explained that Japan adopted DT of GM corn and cotton with familiar traits in 2014 and 2019, respectively. He also introduced that 7 GM corn events have already been accepted for DT until today. As the future prospect, Dr. Hiratsuka mentioned the possibility to expand the scope for accepting DT beyond GM corn and GM cotton with familiar traits with scientific rationale and accumulated evidence.

From Africa, Douglas Miano, University of Nairobi, made a presentation entitled “Evolving dialogue and policy considerations on biosafety data transportability for advancing agricultural biotechnology in Africa.” In Africa, the number of countries with functional Biosafety frameworks increased from 6 to 11 between 2011 and 2022. However, even though there are unpublished reports of the use of DT in some African countries like Ghana and Nigeria, widespread use of DT has not been adopted yet, and currently CFT is required for each country planning to cultivate GM crops. In other regions, CFT is usually conducted for 1 year, but in Africa each country requires an average of about 3 years of CFT. Even though useful CFT data has already been available in some other countries, it is not possible to transport that data, resulting in additional time required for obtaining approval of commercialization of GM crops, making it difficult for African farmers to access this innovation. In the presentation, Dr. Miano referenced the African Union Development Agency-NEPAD (AUDA-NEPAD) which has a flagship biosafety program that was established to support Africa Union (AU) member states on matters of biosafety regulation and to facilitate technical cooperation among them. In recent times, AUDA-NEPAD, Michigan State University (MSU) and Bayer Crop Science established a Science Fellowship Program to design and implement research on existing and potential barriers to the transportability of biotech efficacy data towards timely and cost-effective decision making in Africa. This ongoing research (Miano et al., 2023) explores pragmatic, science-based and fit-for-purpose solutions to help overcome these barriers. By adopting DT, this group is aiming to eliminate redundancies in local (efficacy) testing requirements by leveraging existing data.

From Argentina, Facundo Vesprini, Bayer CropScience, Argentina, made a presentation entitled “Transportability of conclusions from Confined Field Trials.” He presented a theoretical experience exploring the transportability of conclusion of the ERA from Brazil to Argentina, applied to a GM bean which was developed by Brazilian Agricultural Research Enterprise (EMBRAPA), that confers resistance to the Golden Mosaic Virus. This exercise has already been published in 2019 and tests the transportability of conclusions from EMPRAPA 5.1 field trials to bean growing regions in Argentina (Vesprini et al., 2020). To assess the transportability of conclusions of agro-phenotypic (for ERA) and compositional studies (for food and feed safety assessment) carried out in Brazil, three main criteria were established: “appropriate experimental design and methodologies,” “relevance and consistency of measured endpoints across studies” and “diversity of environmental conditions selected for the CFT within the crop production zones.” It is worth noting that similarity of agroclimatic conditions is not included in the criteria. However, it was mentioned that if plausible risk hypotheses were to be identified in a particular environment, local CFT in Argentina may be required, or similarity of climatic conditions become relevant to address the concerns at the particular environment with the available studies. Given the criteria on DT described above, the conclusions of the agro-phenotypic and compositional studies conducted in Brazil were considered transportable to Argentina.

## 4 A risk hypothesis-based approach for DT

To conclude the session, a risk hypothesis-based approach for DT developed in advance by the authors, was introduced (Graphic abstract). For this approach, it is recommended to conduct an ERA with existing information related to the receiving environment, the introduced trait and the biology of host crop, in addition to existing CFT data, when available, before asking for local CFT in other countries by default. In this case, existing CFT data should meet the following two conditions: a) testing should be well-designed comparative study; b) testing should be of an adequate scale, and multiple sites under diverse environmental conditions should be selected. If the previously collected CFT data meets these conditions, regulatory authorities should consider requesting local specific CFT only when a plausible risk hypothesis specific to the receiving environment (such as biological factors, climatic conditions or others) is identified.

This risk hypothesis-based approach for DT introduced during discussion of this parallel session is not entirely new but was developed by integrating the DT criteria and conditions already proposed by several authors during the session. Specifically, this approach is structured around a concept of the ERA based on problem formulation as proposed by Anderson et al, (2021), DT of the data collected in well-designed CFT that test a clear risk hypothesis proposed by Bachman et al. (Bachman et al., 2021) and with transportability of conclusions from confined field trials proposed by Vesprini et al. (2020). These first two papers played a central role in structuring Dr. Simmons’s session and are also part of a “Special Issues on Genetically Modified Organisms” (Molins, 2021). This special issue contains seven reports developed by CropLife International (CLI) to contribute to the development of a scientific and internationally harmonized regulatory system based on 25 years of experience since the start of commercial GM crop cultivation. Two of the seven reports summarize recommendations regarding the ERA. Anderson et al. (2021) explains that the ERA should be based on problem formulation and only require relevant data. Bachman et al. (2021) describes the conditions for DT within the framework of problem formulation. These papers conclude that if no biologically relevant differences between a GM plant and its conventional counterparts are observed in one country or region, data from well-designed studies can be transportable for ERA to another country regardless of agroclimate zone.

The paper (Vesprini et al., 2020) introduced by Eng. Vesprini also recommends local CFT, only if there is a risk hypothesis identified for the GM crops in a particular environment, and pointed out that “appropriate experimental design and methodologies,” “relevance and consistency of measured endpoints across studies” and “diversity of environmental conditions in CFT locations within the crop production zones” are the three key criteria proposed for transportability of conclusion which are basically the same idea as the risk hypothesis-based approach for DT.

Dr. Roberts introduced a paper written by Garcia-Alonso et al. (2014) which propose four conditions for practical DT. One of these criteria is that evaluators must have scientific justification for the use of CFT data from other places, and one way provide this justification is to demonstrate similarity in agroclimate (Melnick et al., 2023). As previously mentioned, he emphasized that the concept of similarity of agroclimatic zone may be helpful for rational selection of CFT sites, but it is only one way to demonstrate that the environment where data was collected is relevant for consideration during risk assessment, not a requirement for DT.

As introduced by Dr. Hiratsuka, Dr. Miano and Eng. Vesprini in this session, there are different degree of adoption of DT, depending on factors like the experience with ERA process for GM crops, public acceptance of GM crops in each country and region, and others. Even in countries that have accumulated experience with the ERA of GM crops, one of the reasons for low adoption of DT is the idea that GM crops will grow differently in different growing environments. In such case, it is important to recognize the purpose of CFT for the ERA, that is, not to characterize GM crops in as much detail as possible in each of different environmental conditions. Rather, the purpose of CFT for GM crops is to identify whether any unintended and adverse changes occurred related to the ERA assessment endpoints by comparing the GM crop to the conventional crop under highly controlled testing conditions (Anderson et al., 2021).

Many papers comparing the results of CFT conducted in different countries and regions have been published and confirmed that no unintended or adverse changes occurred related to the ERA assessment endpoints in GM crops which do not have unique risk hypothesis in the receiving environment. Clawson et al. (2019) shared the results of agronomic characterization of GM corns performed in five regions (Argentina, Brazil, Mexico, Pakistan, and the United States) from 2004 to 2014. It was demonstrated that risk assessment outcomes from agronomic characterization of the 3 GM corn events (MON 89034, NK603, and MON 89034 × NK603) were consistent across multiple global regions. Additionally, Nakai et al. (2015) compared selected plant characteristics data which related to weediness potential from the CFT performed in Japan and multi-locations in the US for already approved 3 GM corn events (LY038, MON 89034, and MON 87460), expressing diverse traits such as nutritional improvement, lepidopteran insect-protection and drought tolerance, respectively. The study showed the differences related to weediness potentials that were not detected in the US CFT were similarly not detected in the Japan CFT for all of the 3 GM corn events. Finally, Matsushita et al. (2020) compared agronomic data from the CFT performed in Japan and multi-locations in the US for already approved 11 GM soybean events and demonstrated the similarity of results obtained in Japan and the US.

As described above, existing conditions for DT that have already been advocated to date have fallen into three categories. The first is Agroclimate similarity-based approach, as introduced by Dr. Robert. The second is Familiarity-based approach, as introduced by Dr. Hiratsuka from Japan and the third is Risk hypothesis-based approach, as introduced by Dr. Simmons and Eng. Vesprini. While each condition maintains scientific validity, a great deal of scientific knowledge has accumulated over more than 20 years, including the result of CFT conducted in various countries and regions subsequent to some of conditions of DT were proposed. In this context, the authors, who had previously advocated different DT conditions, convened for this DT parallel session. After considering the latest scientific insights, they collectively endorsed the Risk hypothesis-based approach as one of the most scientifically valid DT conditions currently available.

Based on the accumulated evidence, the discussion of this parallel session was concluded with an agreement that the proposed risk hypothesis-based approach for DT is scientifically reasonable. The authors believe that the results of local confirmatory CFT, accumulated over the past 20 years and the science-based criteria established for transportability, will contribute the discussions in countries and regions contemplating the adoption of DT in the future, even for GM crops with new traits.
